# The Effect of Exercise on Life Quality and Depression Levels of Breast Cancer Patients

**DOI:** 10.31557/APJCP.2021.22.3.725

**Published:** 2021-03

**Authors:** Mensure Aydin, Elif Kose, Ilhan Odabas, Bergun Meric Bingul, Deniz Demirci, Zeki Aydin

**Affiliations:** 1 *Physical Education and Sport, Halic University, Istanbul, Turkey. *; 2 *Department of Public Health, Sakarya University School of Medicine, Sakarya, Turkey. *; 3 *Faculty of Sport Sciences, Kocaeli University, Kocaeli, Turkey. *; 4 *Faculty of Health Sciences, Uskudar University, Istanbul, Turkey. *; 5 *Darica Farabi Training and Research Hospital. Department of Nephrology, University of Health Sciences, Kocaeli, Turkey. *

**Keywords:** Breast cancer, depression, exercise therapy, quality of life

## Abstract

**Introduction:**

The aim of this study is to determine the effects of aerobic and stretching exercises on quality of life and depression levels of breast cancer patients.

**Methods:**

A total of 48 women (mean age 45.0±2.2 years) who were previously diagnosed with breast cancer and completed their treatment with no metastasis, were included in the study. Of these, 24 women who received the exercise program were assigned as the study group, while the remaining 24 women who did not receive the exercise program were assigned as the control group. The study group received a 12-week aerobic exercise program at the fitness club and home-based resistance exercise program designed by a sport scientist at the doctoral level. The control group was encouraged to maintain their normal level of physical activity and exercise habits throughout the study. The WHOQOL-BREF, EORTC-QLQ-C30 quality of life assessments and Beck depression inventory (BDI) were used to evaluate quality of life and the severity of depression before and after 12-week exercise programs.

**Results:**

EORTC QLQ-C30 scoring showed that in the study group aerobic exercise positively impacted quality of life on the functional scales (physical p=0.001, role p=0.039, emotional p=0.031, social functioning p=0.010) and symptoms (fatigue p=0.001, pain p=0.001, sleep disturbance p=0.038 and financial impact p=0.015). WHOQOL-BREF assessment areas, (general p=0.001, physical p=0.02, mental p=0.001 and social health p=0.017) relationships also improved as a result of exercise. BDI showed that severity of depression in the study group decreased significantly (p=0.001).

**Conclusion:**

This study showed that aerobic and resistance exercises improved quality of life and decreased depression levels of women who previously received breast cancer treatments.

## Introduction

During the treatment of breast cancer, physical health deteriorates due to illness and treatment related physiological changes such as inadequate oxygen in tissues (i.e. anemia) or inadequate blood support to muscles (i.e. cardiotoxic effects of cancer treatments) (Antunes et al., 2019; Do et al., 2015). Despite the established efficacy of breast cancer treatments such as chemotherapy and radiation, they have adverse effects on patients’ cardiovascular, metabolic, and quality-of-life (QoL) outcomes (Gavric and Vukovic-Kostic, 2016). In addition, because of the changes occurring in all stages of treatment from the time of diagnosis, depression is observed in patients alongside with the emotional and behavioral reactions. “Awareness of being diagnosed with cancer,” is a state that obviously threatens the well-being of a person (Emery et al., 2009; Rey-Villar et al., 2017). The majority of cancer patients enter into process of depression after learning of the diagnosis for the first time or as a reaction to worsening and progression of the disease. Rather than the risks that are experienced during the treatment period, fear of death increases the level of anxiety and depression by detaching the patient from life (Ramachandra et al., 2009; Bozo et al., 2019). As a result of the contraindications related to the treatment, reductions in aerobic capacity, fatigue, and loss of muscle strength lead to depression and reduce the quality of life (Gavric et al., 2016; Heydarnejad et al., 2011). A healthy lifestyle that includes regular physical activity is essential to minimize the psychological and physical side effects of treatment (Yaw et al., 2014).

With the advancement in modern medicine, early diagnosis of cancer, given medications, radiation and surgical interventions increase the survival time of the patients, but the quality of life in relation to health has become more important (Sawyer, 2014). Recent studies emphasize that exercise plays a crucial role in decreased physical performance in patients receiving cancer treatment. It is stated that a low to moderate intensity exercise program applied to patients alleviate several symptoms of the disease and can be considered as the part of the rehabilitation (An et al., 2020; Chen et al., 2009). Various studies showed that regular exercise program applied to patients during or after the cancer treatments, improved the quality of the life as a result of obtaining ideal body weight, cardiopulmonary fitness, decrease in fatigue sense, neuromuscular integrity, increase in muscle strength and elasticity, and improvements in psycho-social well being (Do et al., 2015; Valenti et al., 2018; Zhang et al., 2019).

Effort to promote the QOL in breast cancer patients is considered as one the most important topics in women’s health care (Shandiz et al., 2017). Zaker et al., (2019)exercise for breast cancer patients; they suggested that it might have a positive effect on their compliance with the complications of the disease and their treatment It is important to know, during breast cancer chemotherapy, what type of exercise program aiming to support treatment and to increase the quality of life of the patients should be given for how long and at which stage of the treatment (Chen et al., 2009; Muktezanı et al., 2014). During cancer treatments, when the relationship of the exercise and physical fitness with cardiorespiratory, muscular fitness and body composition is examined, it is shown that exercise regulates cardiovascular fitness (moderate evidence), while resistance exercise alone increases the muscular strength and conserves body composition at a desired level (Aly et al., 2017; Mutrie et al., 2007).

It is seen in the literature that aerobic and resistance exercises are commonly studied, however, while there are studies looking at combination of two exercises, few of these were seen to study home-based resistance exercises. Moreover, five days or more times a week and safe and feasible exercise programs are found to be less common in the literature (Thorsen et al., 2005).

Exercise is considered to be the most effective method in the cancer rehabilitation, and exercise programs (aerobic and/or other applicable programs) are thought to be more effective when performed at home (Sweegers et al., 2019). As a result from this study, it is expected that being away from the hospital atmosphere, in a safe and hygienic home environment, using their own equipment and doing exercise everyday, would enhance subjects’ quality of life and reduce depression. 

The aim of this study is to determine whether 60-minute aerobic and home-based resistance exercise programs have any effect on the quality of life and depression levels of the breast cancer patients who completed their treatments (surgery, chemotherapy, radiotherapy) and started their routine controls.

## Materials and Methods

The study was selected from individuals who were treated in Kocaeli University Medical Faculty Hospital, Oncology Clinic and volunteered to participate in the study. Our study consisted of patients who were treated in hospital and who came for routine control who could follow the program. The next patient was randomized. Inclusion criteria; Patients who had undergone partial or total breast surgery for breast cancer and who had not developed distant organ metastases who agreed to participate in an exercise program were included in the study. Exclusion criteria; Those with distant organ metastasis and health problems who cannot do the exercises to be applied; patients with advanced heart failure, advanced liver failure, severe anemia (Hb less than 8 g/dl), physically disabled, mentally impaired patients and patients who do not accept to work. This is an experimental, comparative research to investigate the effect of aerobic and resistance exercises on the quality of life and depression levels of the breast cancer patients who completed their treatments and started their routine controls. Before initiating the study, all the participants were informed about the study procedure. A written informed consent was obtained from each participant and they were instructed to stop exercise in case of any adverse event such as excessive fatigue, dizziness, feeling of fainting, and/or increased lymphedema. The study protocol was approved by the Ethics Committee (Ethics committee document number: 2018/44).

Between September 2017 and March 2018, a total of 48 women volunteers (mean age 45 .0±2.2 years), who were previously diagnosed with breast cancer and completed their treatment with no metastasis, were included in the study. Of these, 24 women who received the exercise program were assigned as the study group, while the remaining 24 women who did not receive the exercise program were assigned as the control group. 

Demographic data and medical history were obtained from all volunteers prior to study measurements and exercise. In order to measure the effect of exercise on quality of life and depression, before and after exercise, two measurements were performed on both groups with 12-week interval using questionnaires.

*Exercise Procedures*

The study group received a 12-week aerobic exercise in the fitness club and home-based resistance exercise program designed by a sport scientist at the doctoral level. The control group was encouraged to maintain their normal level of physical activity and exercise habits throughout the study. 

*Aerobic Exercise*

The aerobic exercise program is performed 3 days a week, 50-minute bouts at 50-60% of their maximum heart rate (HRmax). 

The target heart rates were calculated as moderate (50-60% of the heart rate or 11-14 on the Borg Scale) target heart rate according to Karvonen method (Ferrer et al., 2011). Treadmills and exercise bikes at fitness center were used for the aerobic exercises. The aerobic exercise program included 10 minutes of warming up, 30 minutes of walking and cycling, and 10 minutes of cooling down performed 3 days a week. 

As the exercise capacity of the participants increased, their speed and working time were also increased. The exercise was terminated in the event of excessive fatigue, dizziness, feeling of fainting and/or increased lymphedema.

*Home Exercise*

The home-based portion of the exercise program consisted of resistance training that was to be carried out at least twice a week for 60 minutes. For resistance exercises, 10-minute warm up, followed by 40-minute leg and hip workout by using elastic band and ball, and 10-minute cool down have been performed.

A 20-page booklet, titled “Exercises for the Oncology Patients,” was provided to the volunteers in the study. This booklet has been given to both groups. The booklet included information and instructions on engaging in regular exercise while at home. During the training session, a sports training specialist demonstrated in practice the exercise program and checked whether the exercises were performed right. 

*Data Collection Tools*

World Health Organization Quality of Life Scale (WHOQOL-BREF), EORTC-QLQ-C30 Scale and Beck depression inventory (BDI) were used to evaluate quality of life and the severity of depression before and after 12-week exercise programs. General quality of life of the patients World Health Organization Quality of Life Scale Turkish short form (WHOQOL-BREF TR) (Simsir Atalay et al., 2011), and the EORTC-QLQ-C30 scale, which measures the quality of life in patients with cancer, which is widely used worldwide and developed by EORTC (Gürler et al., 2014).

World Health Organization Quality of Life Scale is a self-report questionnaire consists of 24 items including physical health (7 items), psychological (6 items), social relationships (3 items), and environment (8 items) (Li et al., 2004). 

The purpose of the EORTC QLQ-C30 questionnaire is to measure cancer patients’ physical, psychological and social functions. The questionnaire comprises 30 items, 24 of which are aggregated into 9 multi-item scales: 5 functioning scales (physical, role, cognitive, emotional, and social); 3 symptom scales (fatigue, pain, and nausea/vomiting); and 1 global health status scale. The remaining six single items assess symptoms of dyspnea, appetite loss (AP), sleep disturbance, constipation, diarrhea, and financial impact. Both study and control groups used version 2 of the QLQ-C30, and a standard scoring was applied to the scales (Musoro et al., 2019).

Beck depression inventory was developed in 1961 by Aaron T Beck and revised in 1979 by Beck (1961). The purpose of this inventory is to assess the severity of depression and it consists of 21 items measuring cognitive, emotional, motivational, and somatic symptoms of depression. For each item, subjects are expected to choose one of the four response alternatives to describe how they felt within the previous week. The scores of each item ranges from 0 to 3, and higher scores indicate higher levels of depressive symptoms. The investigator used the modified inventory divided into three grades: minimal range, 14–19 is mild, 20–28 is moderate, and 29–63 is severe) (Bozo et al., 2019). 

*Statistical Analysis*

Statistical analyses were performed using Statistical Package for the Social Sciences (SPSS 22) software. Numerical variables that exhibited normal distribution were given as mean ± standard deviation; whereas those having non-parametric data as median and interquartile range (IQR); the categorical variables as frequency and percentage. Of numerical variables with normal distribution; for two-group comparisons student t-test and paired sample t test were used. Of numerical variables with abnormal distribution; for two-group comparisons Mann-Whitney U and Wilcoxon test test was used. For all statistical analyses p value < 0.05 was considered to be statistically significant.

## Results

A total of 48 women (mean age 45.0±2.2years) who were previously diagnosed with breast cancer and completed their treatment with no metastasis, were included in the study. Of these, 24 women who received the exercise program were assigned as the study group, while the remaining 24 women who did not receive the exercise program were assigned as the control group.

*Demographic features*

Age: Exercise group and the control group, the most patient age group was 55-60 years (37.5%)

Educational status: Most of the Exercise group are high school graduates (33.3%), while the control group (29.2%) is primary school graduates.

Occupation: It was observed that both groups in the study were housewives (exercise group 33.3%, control group 29.2%).

Economic status: The income level of the exercise group is between 600-750 $ (41.7%), and the control group is between 450-600 (29.2%) and 600-750 $ (29.2%).

Total number of children: Exercise group has 2 children (41.7%) and control group has 3 children (37.5%) (Table 1).

*WHOQOL-BREF quality of life scale*

A significant difference has been found in all parameters of the group participating; general health [50.0 (37.5-59.4) vs 75.0 (62.5-75.0), p=0.001], physical halth [50.0 (39.3-53.6) vs 57.1 (50.9-63.4), p=0.002], psychological health (55.5±15.0 vs 65.4±8.7, p=0.001), social health (50.3±22.2 vs 62.1±15.7, p=0.017), environment health (56.9±17.6 vs 70.3±11.2, p=0.001) in the exercise program (Table 2). In the study, in the study and control group comparison; There was a significant difference in general health (p=0.001), physical health (p=0.001), psychological health (p=0.009), social health (p=0.016) environmental health (p=0.002) parameters (Table 3).

*EORTC-QLQ-C30 quality of life scale*

Functional parameters of the group participating in the exercise program; a significant difference has been found in physical (p=0.011), role (p=0.039), emotional (p=0.031), social areas (p=0.010). In the symptom parameters of the group participating in the exercise program, a significant difference has been found among fatigue (p=0.001), pain (p=0.001), sleep disturbance (p=0.038) and financial impact (p=0.015). Functional parameters of control group; a significant difference has been found in role (p=0.011), cognitive (p=0.07), and social areas (p=0.048). A negative significant difference has been found in the quality of life area (p=0.001). There has been a decline in quality of life. In the control group symptom parameters, a significant difference has been determined in pain (p=0.003), sleep disturbance (p=0.040), appetite loss (p=0.001) areas. A negative significant difference has been found in fetigue area (p=0.001) (Table 4).

In the study, functional physical (p=0.001), social (p=0.009) and quality of life (p=0.001) parameters were significantly different in favor of the exercise group, while a significant difference in the cognitive parameter was found in favor of the control group. In terms of symptoms nause (p=0.038), sleep disturbance (p=0.033), loss of appetite (p=0.001) and financial impact (p=0.004) of the parameters is significantly different in favor of the exercise group, the control group, but there is a significant difference in favor of loss of appetite in the parameter (Table 5).

*Beck depression inventory scale*

The distribution and comparison of Beck Depression Scale scores of exercise and non-exercise groups are shown in Table 6. A significant difference has been found in the depression scores of the study group before and after the exercise program (p<0.001). The result for the depression levels of the study group and control group was found to be significantly different from each other (p<0.001) (Table 7).

**Table 1:** Demographic Features

	Exercise Group		Control Group	
	n	%	n	%
Age	24		24	
40-45	3.0	12.5	4.0	16.7
45-50	5.0	20.0	6.0	25.0
50-55	7.0	29.2	6.0	25.0
55-60	9.0	37.5	8.0	33.3
Education status				
Literate	1.0	4.2	2.0	8.3
Primary school	5.0	20.8	7.0	29.2
Secondary school	5.0	20.8	5.0	20.8
High school	8.0	33.3	6.0	25.0
University	4.0	16.7	4.0	16.7
University and above	1.0	4.2	0.0	0.0
Occupation				
Housewife	8.0	33.3	7.0	29.2
Officer	5.0	20.8	5.0	20.8
Employee	6.0	25.0	7.0	29.2
Retired	5.0	20.8	6.0	25.0
Income ($)				
300-450	3.0	12.5	5.0	20.8
450-600	6.0	25.0	7.0	29.2
600-750	10.0	41.7	7.0	29.2
750 above	5.0	20.8	5.0	20.8
Total number of children (24)				
1	1.0	4.2	2.0	8.3
2	10.0	41.7	7.0	29.2
3	8.0	33.3	9.0	37.5
4 +above	5.0	20.8	6.0	25.0
Total	24.0	100.0	24.0	100.0

**Table 2:** Evaluation of the WHOQOL Pre-Test and Post-Test of the Groups Participating and not Participating in the Exercise Program

	Exercise group		p	Control group		p
WHOQOL	Pretest	Posttest		Pretest	Posttest	
General Health (IQR)**	50.0 (37.5-59.4)	75.0 (62.5-75.0)	<0.001	46.9±14.8	46.9±14.8	0.908
Physical Health (IQR)**	50.0 (39.3-53.6)	57.1 (50.9-63.4)	0.002	45.0±12.7	45.0±12.7	0.912
Psychological*	55.5±15.0	65.4±8.7	0.001	55.2±15.0	56.1±14.3	0.785
Social Relationship*	50.3±22.2	62.1±15.7	0.017	48.3±21.0	48.3±21.0	0.908
Environment*	56.9±17.6	70.3±11.2	<0.001	56.4±17.4	56.4±17.4	0.908

*, Student t test, mean± standard deviation; **, Mann-Whitney U test, median (IQR: Interquartile range).

**Table 3:** Distribution and Comparison of WHOQOL Scale Scores of Exercise (Study) and non-Exercise (Control) Groups

WHOQOL	Exercise Group	Control Group	tZ	p
General Health*	68.2±12.7	46.87±14.85	-4,279	<0.001
Physical Health**	57.1 (50.9-63.4)	46.4 (36.6-53.6)	-3,304	0.001
Psychological Health*	65.4±8.7	56.1±14.3	2,745	0.009
Social Health*	62.1±15.7	48.3±21.0	-2,400	0.016
Environment Health*	70.3±11.2	56.4±17.4	3,298	0.002

*, Student t test; mean± standard deviation; **, Mann-Whitney U test, median (IQR, Interquartile range).

**Table 4:** Evaluation of the EORTC Scale before and after Intervention in the Exercise and Control Group Participating in the Study

	Exercise Group		T.Z	p	Control Group		T.Z	p
EORTC	Pretest	Posttest			Pretest	Posttest		
Functional								
Physical**	60.0 (46.6-80.0)	83.6 (75.0-93.3)	-3,940	<0.001	60.0 (46.6-80.0)	66.6 (40.0-73.3)	-0.428	0.668
Role**	66.6 (33.3-100.0)	83.3 (66.6-100.0)	-2,066	0.039	66.6 (40.0-73.3)	83.3 (50.0-100.0	-2,558	0.011
Cognitive**	83.3 (66.6-100.0)	83.3 (66.6-100.0	-0.450	0.653	83.3 (66.6-100.0)	58.3 (50.0-83.3)	-2,395	0.017
Emotional**	75.0 (35.4-83.3)	95.8 (52.1-100.0)	-2,163	0.031	75.0 (41.6-83.3)	75.0 (43.7-83.3)	-1,427	0.154
Social**	66.6 (37.5-83.3)	100.0 (70.8-100.0)	-2,568	0.010	66.6 (37.5-83.3)	66.6 (66.6-95.8)	-1,979	0.048
Quality of Life*	46.9±23.7	78.8±14.1	-0.198	0.354	46.8±23.6	33.7±13.8	4,708	<0.001
Symptom								
Fatigue*	58.8±27.8	34.2±18.2	-5,241	<0.001	58.3±27.8	38.4±22.9	3,723	0.001
Nause**	16.6 (0.0-29.2)	0.0 (0.0-16.6)	-1,248	0.212	16.6 (0.0-29.2)	16.6 (0.0-33.0)	-1,403	0.161
Pain**	250.0 (20.8-95.8)	33.3 (16.6-45.8)	-3,376	0.001	50.0 (20.8-95.8)	33.3 (20.8-50.0)	-2,921	0.003
Dyspnea**	0.0 (0.0-33.3)	0.0 (0.0-33.3)	-0.284	0.776	0.0 (0.0-33.3)	3.3 (0.0-58.3)	-1,706	0.088
Sleep Disturbance**	33.3 (8.3-66.6)	33.3 (0-58.3)	-2,072	0.038	33.3 (8.3-66.6)	66.6 (33.3-91.7)	-2,054	0.040
Appetite Loss**	0.0 (0.0-33.3)	0.0 (0.0-25.0)	-1,063	0.288	0.0 (0.0-33.3)	66.6 (33.3-66.6)	-3,675	<0.001
Constipation**	0.0 (0.0-33.3)	0.0 (0.0-33.3)	-0.977	0.329	0.0 (0.0-33.3)	0.0 (0.0-0.0)	-1,095	0.273
Diarrhea**	0.0 (0.0-33.3)	0.0 (0.0-0.0)	-1,155	0.248	0.0 (0.0-33.3)	0.0 (0.0-33.3)	-0.812	0.417
Financial Impact**	33.3 (0.0-66.6)	0.0 (0.0-33.3)	-2,441	0.015	33.3 (0.0-66.7)	33.3 (8.3-66.6)	-0.372	0.710

*, Student t test; mean± standard deviation; **, Mann-Whitney U test, median (IQR, Interquartile range)

**Table 5:** Distribution and Comparison of EORTC QLQ-30 Scale Scores of Exercise and non-Exercise (Control) Groups

EORTC QLQ-30	Exercise Group	Control Group	p
Functional			
Physical	83.6±11.1	59.7±21.7	<0.001
Role	77.8±22.3	75.0±25.1	0.732
Cognitive	79.9±22.5	61.8±29.7	0.029
Emotional	80.2±24.8	67.0±26.4	0.051
Social	87.5±19.2	71.5±22.8	0.009
Quality of life	78.9±14.1	33.7±13.8	<0.001
Symptom			
Fatigue (IQR)	33.3 (22.2-44.4)	33.3 (22.2-63.8)	0.777
Nause (IQR)	0.0 (0.0-16.6)	16.7 (0-33.3)	0.038
Pain (IQR)	33.3 (16.7-45.9)	33.3 (20.8-50.0)	0.184
Dyspnea (IQR)	0 (0-33.3)	33.3 (0-58.3)	0.300
Sleep disturbance (IQR)	33.3 (0-58.3)	66.6 (33.3-91.7)	0.003
Appetite loss (IQR)	0 (0-25.0)	66.6 (33.3-66.7)	<0.001
Constipation (IQR)	0 (0-33.3)	0 (0-0)	0.393
Diarrhea (IQR)	0 (0-0)	0 (0-0)	0.150
Financial impact (IQR)	0 (0-33.3)	33.3 (8.3-66.7)	0.004

IQR, Interquartile range

**Table 6:** Distribution and Comparison of Beck Depression Inventory Scale Scores of Exercise and non-Exercise (Contrrol) Groups (Minimal Range, 14–19 is Mild, 20–28 is Moderate, and 29–63 is Severe

	Exercise Group						Control Group					
Depression	Before		After		t.Z	p	Before		After		t.Z	p
	n	%	n	%			n	%	n	%		
Minimal	4	16.7	16	66.7			3	12.4	5	4.1		
Mild	10	41.7	6	25.0			11	45.7	9	37.5		
Moderate	7	29.2	2	8.0	-3,630	<0.001	7	29.2	8	33.3		
Severe	3	12.5	-	0			3	12.5	2	8.3	-1,414	0.157
BECK Scores (IQR)	15.5 (11.0-20.7)		5.0 (1.2-11.7)		5,300	<0.001	15.5 (11.0-20.7)		15.0 (10.2-22.5)		-0.594	0.558

IQR, Interquartile range

**Table 7:** Distribution and Comparison of Beck Depression Inventory Scale Scores of Exercise and Control Groups

Depression	Study Group		Control Group		Z	p*
	n	%	n	%		
Minimal	16	66.7	5	20.1		
Mild	6	25.0	9	37.5	-3.413	0.001
Moderate	2	8.5	8	33.3		
Severe	-	-	2	8.0		
BECK Scores (IQR)	5.0 (1.2-11.7)		15.0 (10.2-22.5)		-3.893	<0.001

IQR, Interquartile range

## Discussion

The importance of exercise for cancer patients is commonly emphasized. In the literature, it is stated that regular exercise improves physical function, aerobic capacity, strength and flexibility as well as boosts the immune system of the cancer patients. In addition, the improvement of the physical capacity of cancer patients with exercise provides psychologically better feeling reducing stress, depression and anxiety. What is more, exercise reduces the length of hospitalization (or duration of hospital stay) by increasing bone mineral density, and alleviating symptoms of nausea, pain, sleep disorder, and diarrhea in cancer patients (Aly et al., 2017).

It is known that cancer diagnosis and treatment commonly increase the depression levels of the patients (Ramachandra et al., 2009). Having uncertainty about the treatment, physical symptoms, risk of recurrence, fear of death, change in identity of womanhood, sexuality and body image, difficulties in daily activities, family problems and insufficiency of psychological support lead to serious mental/psychological problems in breast cancer patients and this situation triggers depression by creating noncompliance to cancer and sense of despair (Bardwell et al., 2004).

Patsou et al., (2017) studied the effects of physical activity on depressive symptoms during breast cancer survivorship, which is a meta-analysis of randomized control trials between 2011 and 2016 with 1701 breast cancer patients. The study concluded that there were significant differences between the exercise and non-exercise groups (in terms of depressive symptoms) so that an applicable exercise program should be given to breast cancer patients after the treatment. Our study also showed that depression levels of the study group decreased (p=0.001) remarkably as a result of the exercise while depression levels increased in control group who did not perform any exercise.

EORTC-QLQ-C30 scale is one of the widely used assessments to measure quality of life of the cancer patients. Canário et al., (2016) studied physical activity, fatigue and quality of life of 215 breast cancer patients aged between 40-60. They showed that quality of life of the physically active subjects increased and most non-active subjects were fatigued (72.1%) while physically active women showed lower symptoms of fatigue. It was also observed in the same study that according to EORTC QLQ-C30 assessments, active subjects scored higher for all scales, and their quality of life scores were better than the non-active group. 

Results of the 8-week multimodal rehabilitation program for 62 breast cancer women also showed that significant differences of nausea, fatigue and dyspnea parameters assessed by EORTC-QLQ-C30 scale led to general health, physical, role and social functioning parameters to be also affected significantly for the women who started the program in the beginning of the treatment (n=32) compared to the women who started the program after their treatment (n=30) (Do et al., 2015). A combined resistance and aerobic exercise program, similar to our study, applied on the older breast cancer survivors undergoing aromatase inhibitor therapy showed that quality of life parameters such as physical functioning, health, pain, general health sense, vitality, social functioning, fatigue, and sleep disorder were improved significantly (Penttinen et al., 2019). According to the results of EORTC-QLQ-C30 scale in another study, 12-week resistance exercise during breast cancer treatment improved quality of life parameters such as sleep quality, physical fitness, body image and fatigue of the patients (Arab et al., 2016). In agreement with the literature, in our study, functional parameters of the group participating in the exercise program; a significant difference has been found in physical (p=0.011), role (p=0.039), emotional (p=0.031), social areas (p=0.010). In the symptom parameters of the group participating in the exercise program, a significant difference has been found among fatigue (p=0.001), pain (p=0.001), sleep disturbance (p=0.038) and financial impact (p=0.015). Functional parameters of control group; a significant difference has been found in role (p=0.011), cognitive (p=0.07), and social areas (p=0.048) A negative significant difference has been found in the quality of life area (p=0.001). There has been a decline in quality of life. In the control group symptom parameters, a significant difference has been determined in pain (p=0.003), sleep disturbance (p=0.040), appetite loss (p=0.001) areas. A negative significant difference has been found in fetigue area (p=0.001).

QOL of the group receiving conventional physiotherapy accompanied by aerobic exercise was improved significantly when compared to other group receiving conventional physiotherapy alone (p<0.007, t=3.58, d.f.=52), showing that conventional physiotherapy when combined with aerobic exercise is more effective than conventional physiotherapy alone in improving QOL of breast cancer patients (Kulkarni et al., 2013). In our study, WHOQOL-BREF scale evaluation showed that in the study group, general (p=0.001), physical (p=0.002), psychological (p=0.009), social (p=0.016) and environmental health (p=0.002) parameters were positively affected compared to control group. 

In conclusion, our study showed that according to EORTC-QLQ-C30 quality of life scale, exercise positively affected functional parameters (physical, social, and life quality), symptoms (loss of appetite, fatigue, sleep disorder) in breast cancer patients. In patients who were assigned exercise program, significant improvements were observed in several subscale parameters of WHOQOL-BREF quality of life scale, namely, general, physical, and psychological health, social, and environment. According to BDI results, it was observed that depression levels were reduced in the study group. In conclusion, this study showed that aerobic and resistance exercises improved quality of life and decreased depression levels of women who previously received breast cancer treatments. 

## Author Contribution Statement

Prof Dr. Mensure Aydin: The idea for research article hypothesis generation. Assistant of Prof Dr. Elif Kose: Responsblitiy for statistical analysis. Assistant of Prof Dr. Hatice İlhan Odabas: Planning the methods to generate hypothesis. Associate of Prof Dr. Bergun Meric Bingul: Responsibility for exercise practice, management of patients, organising and reporting data. Associate of Prof Dr. Deniz Demirci: Responsibility for conducting literatüre search.Prof Dr. Zeki Aydin: Responsibility for creation of entire or a substantial part of the manuscript
